# Supplementary material for: Epigenetic targeting of bromodomain protein BRD4 counteracts cancer cachexia and prolongs survival
Source: Nat Commun. 2017 Nov 22;8:1707. doi: 10.1038/s41467-017-01645-7 (PMC5700099; doi:10.1038/s41467-017-01645-7)
Supplement: Supplementary file 1 — Description of Additional Supplementary Files [file 41467_2017_1645_MOESM1_ESM.pdf]

Description of Additional Supplementary Files

File Name: Supplementary Data 1
Description: GO categories for BRD4 Chip-seq peaks
