# Supplementary material for: Epigenetic targeting of bromodomain protein BRD4 counteracts cancer cachexia and prolongs survival
Source: Nat Commun. 2017 Nov 22;8:1707. doi: 10.1038/s41467-017-01645-7 (PMC5700099; doi:10.1038/s41467-017-01645-7)
Supplement: Supplementary file 2 — Supplementary Information [file 41467_2017_1645_MOESM2_ESM.pdf]

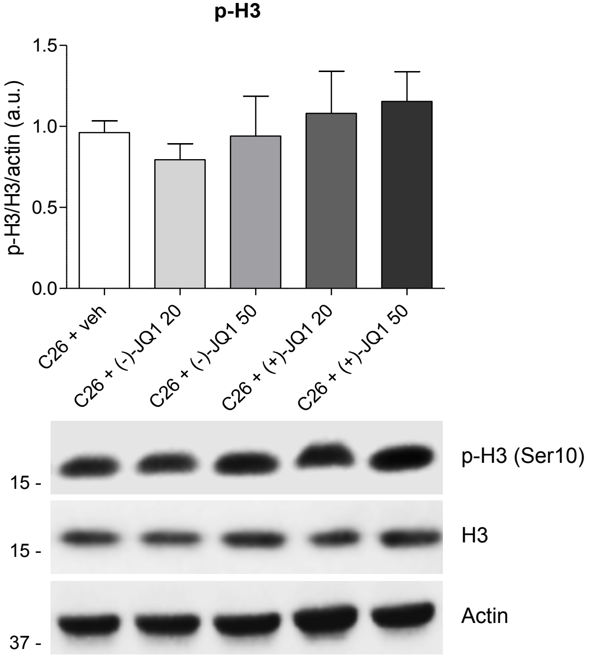
p-H3
p-H3/H3/actin (a.u.)
1.5
1.0
0.5
0.0
C26 + veh
C26 + (-)-JQ1 20
C26 + (-)-JQ1 50
C26 + (+)-JQ1 20
C26 + (+)-JQ1 50
15 -
p-H3 (Ser10)
15 -
H3
37 -
Actin

Supplementary Fig.1 pH3Ser10 phosphorylation is not affected by JQ1 treatment in C26 tumors

p-H3 (Ser10) levels were assessed in C26 tumors by Western blot. 4 animals were used for each experimental group. Data represent means±SD. Statistical analysis was performed by using one-way ANOVA followed by Tukey's post-hoc test.

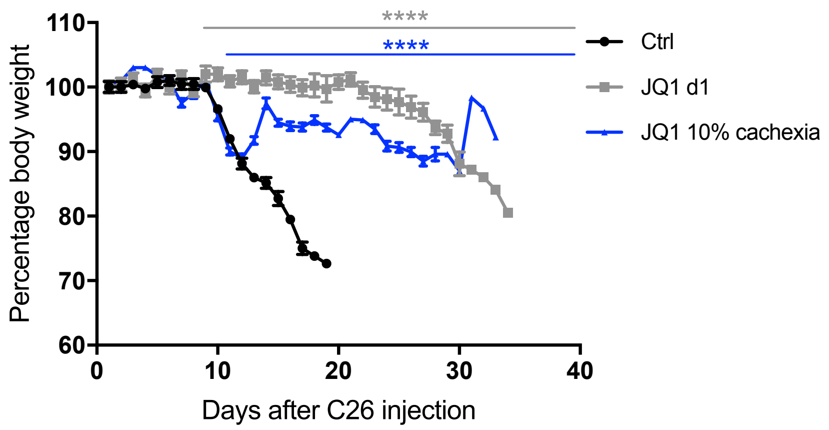
****
****
Ctrl
JQ1 d1
JQ1 10% cachexia
Percentage body weight
110
100
90
80
70
60
0
10
20
30
40
Days after C26 injection

Supplementary Fig.2. Changes in body weights in JQ1-treated mice, in survival experiments

Body weights chart of control and JQ1-treated animals presented in Fig. 2C. Animals per group: n=7. Statistical analysis was performed by using one-way ANOVA. Data represent means±SEM. ****$p<0.0001$.

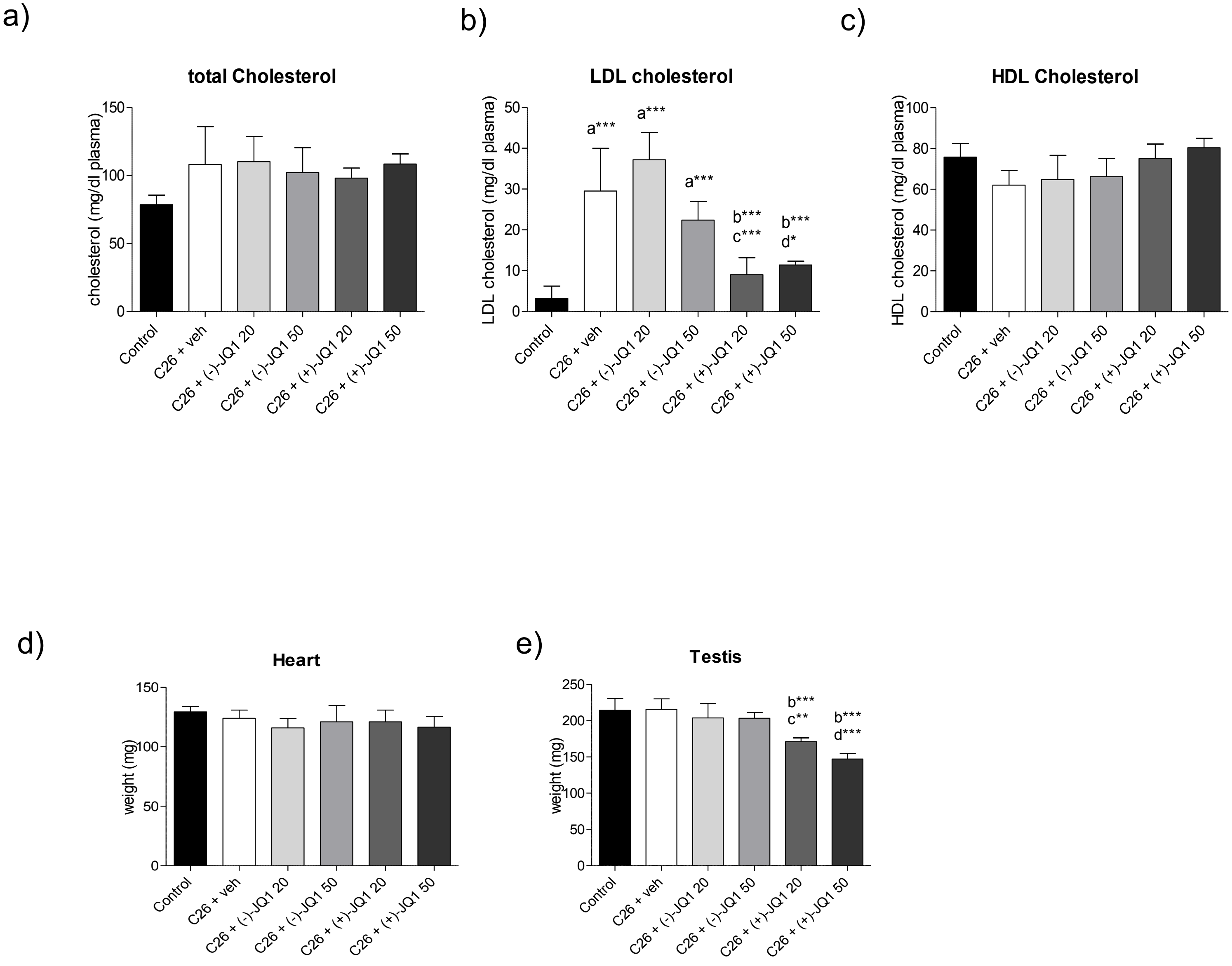
a)
total Cholesterol
cholesterol (mg/dl plasma)
150
100
50
0
Control
C26 + veh
C26 + (-)-JQ1 20
C26 + (-)-JQ1 50
C26 + (+)-JQ1 20
C26 + (+)-JQ1 50
b)
LDL cholesterol
LDL cholesterol (mg/dl plasma)
50
40
30
20
10
0
a***
a***
a***
b***
c***
b***
d*
Control
C26 + veh
C26 + (-)-JQ1 20
C26 + (-)-JQ1 50
C26 + (+)-JQ1 20
C26 + (+)-JQ1 50
c)
HDL Cholesterol
HDL cholesterol (mg/dl plasma)
100
80
60
40
20
0
Control
C26 + veh
C26 + (-)-JQ1 20
C26 + (-)-JQ1 50
C26 + (+)-JQ1 20
C26 + (+)-JQ1 50
d)
Heart
weight (mg)
150
100
50
0
Control
C26 + veh
C26 + (-)-JQ1 20
C26 + (-)-JQ1 50
C26 + (+)-JQ1 20
C26 + (+)-JQ1 50
e)
Testis
weight (mg)
250
200
150
100
50
0
b***
c**
b***
d***
Control
C26 + veh
C26 + (-)-JQ1 20
C26 + (-)-JQ1 50
C26 + (+)-JQ1 20
C26 + (+)-JQ1 50

Supplementary Fig.3. Impact of JQ1 treatment on plasma cholesterol and target tissues

(**a-c**) total, LDL- and HDL-cholesterol levels were measured in plasma samples of control, and C26 tumor-bearing mice treated with vehicle, (-)-JQ1 and (+)-JQ1. 5 animals were used for each experimental condition. Statistical analysis was performed by using one-way ANOVA. Data represent means±SD. *p<0.05; ***p<0.001. “a” indicates statistical significance compared to Control; “b” indicates statistical significance compared to C26+vehicle; “c” indicates statistical significance compared to C26(-)-JQ1 20mg/kg/day; “d” indicates statistical significance compared to C26(-)-JQ1 50mg/kg/day.

(**d,e**) Weight of heart and testis was determined 12 days after C26 cell implantation in C26 tumor-bearing mice treated with vehicle, (-)-JQ1 and (+)-JQ1 (20 and 50 mg/kg/day). Animals per group: n=10. Data represent means±SD. Statistical analysis was performed by using one-way ANOVA followed by Tukey’s post-hoc test. **p<0.01; ***p<0.001. “b” indicates statistical significance compared to C26+vehicle; “c” indicates statistical significance compared to C26(-)-JQ1 20mg/kg/day; “d” indicates statistical significance compared to C26(-)-JQ1 50mg/kg/day.

a)

b)

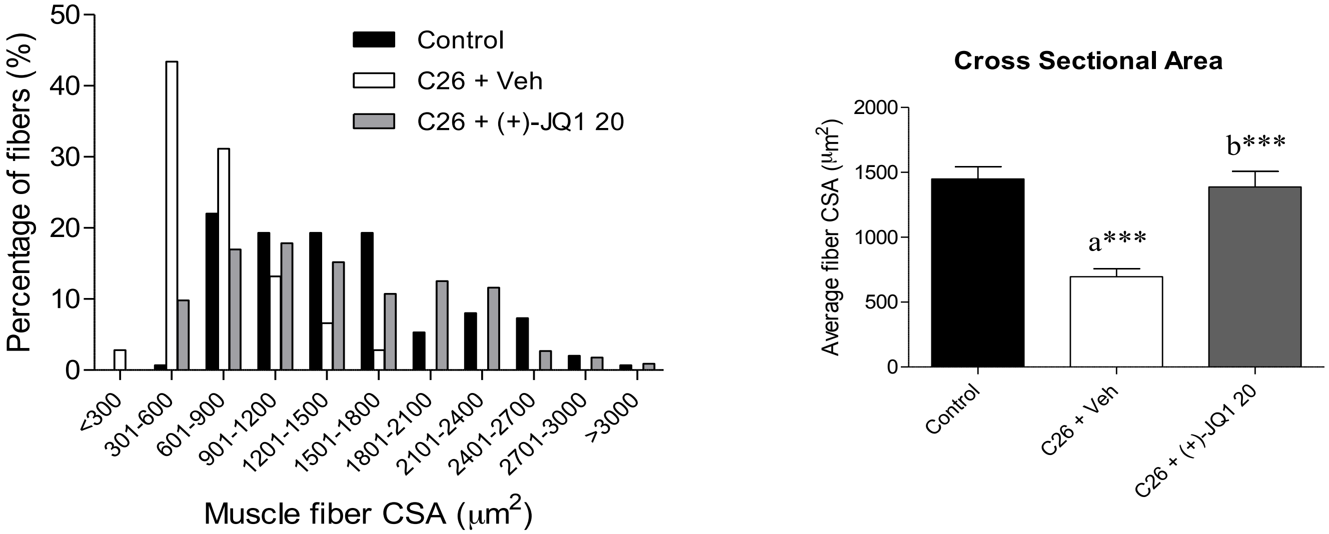

Percentage of fibers (%)
Control
C26 + Veh
C26 + (+)-JQ1 20
<300
301-600
601-900
901-1200
1201-1500
1501-1800
1801-2100
2101-2400
2401-2700
2701-3000
>3000
Muscle fiber CSA (μm2)
Cross Sectional Area
Average fiber CSA (μm2)
a***
b***
Control
C26 + Veh
C26 + (+)-JQ1 20

Supplementary Fig.4. JQ1 administration prevents myofibers size reduction

(**a**) histogram of fiber cross-sectional area (CSA) distribution of TA muscles from control, C26+vehicle, and C26(+)-JQ1.

(**b**) average fiber CSA (average values ± 95% CI), in TA muscles from control, C26+vehicle, and C26(+)-JQ1 animals. Statistical analysis was performed by using one-way ANOVA followed by Tukey's post-hoc test.. ***$p<0.001$; "a" indicates statistical significance compared to Control; "b" indicates statistical significance compared to C26+veh.

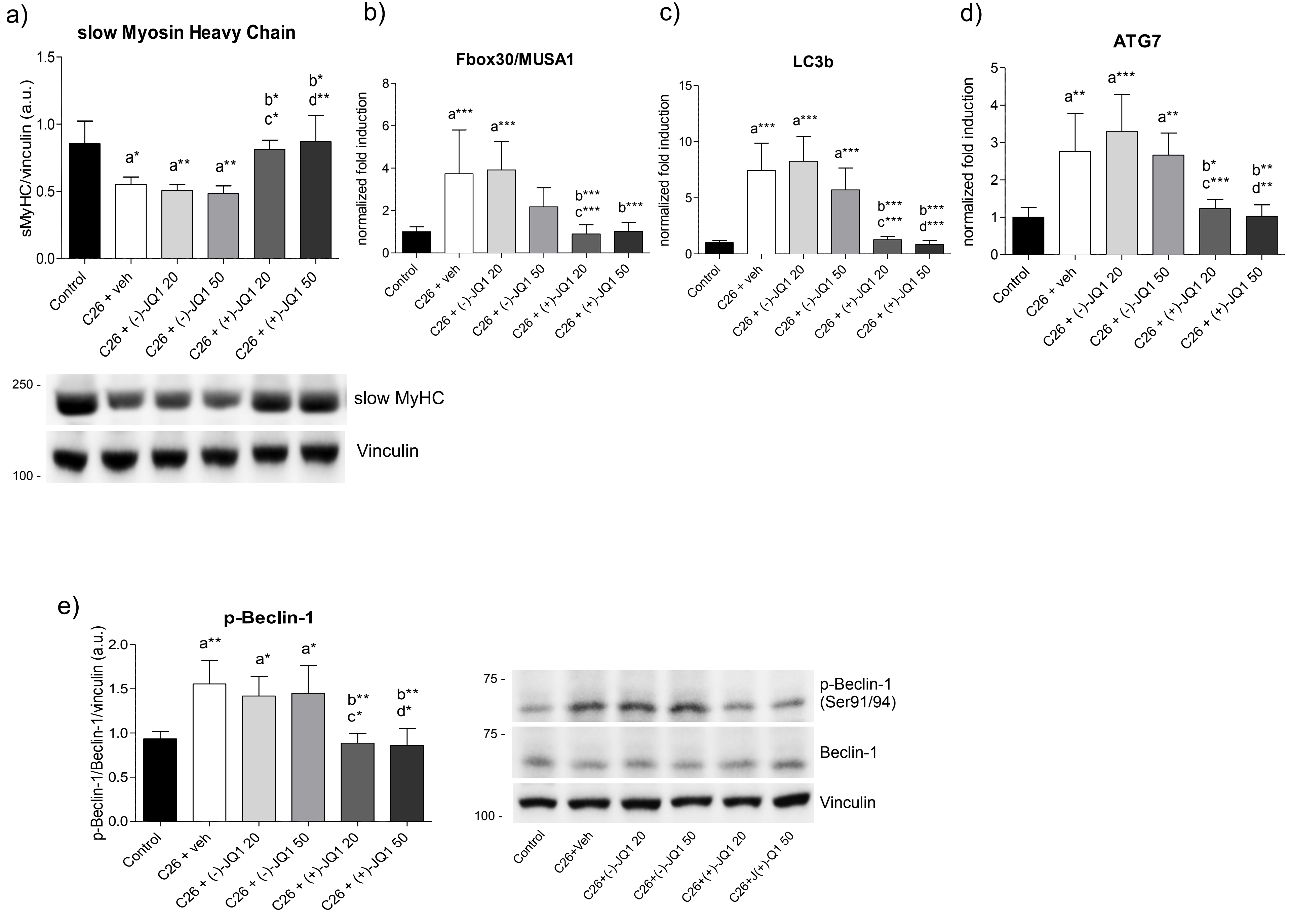

a)
slow Myosin Heavy Chain
sMyHC/vinculin (a.u.)
0.0
0.5
1.0
1.5
a*
a**
a**
b*
c*
b*
d**
Control
C26 + veh
C26 + (-)-JQ1 20
C26 + (-)-JQ1 50
C26 + (+)-JQ1 20
C26 + (+)-JQ1 50
250 -
100 -
slow MyHC
Vinculin
b)
Fbox30/MUSA1
normalized fold induction
0
2
4
6
8
a***
a***
b***
c***
b***
Control
C26 + veh
C26 + (-)-JQ1 20
C26 + (-)-JQ1 50
C26 + (+)-JQ1 20
C26 + (+)-JQ1 50
c)
LC3b
normalized fold induction
0
5
10
15
a***
a***
a***
b***
c***
b***
d***
Control
C26 + veh
C26 + (-)-JQ1 20
C26 + (-)-JQ1 50
C26 + (+)-JQ1 20
C26 + (+)-JQ1 50
d)
ATG7
normalized fold induction
0
1
2
3
4
5
a**
a***
a**
b*
c***
b**
d**
Control
C26 + veh
C26 + (-)-JQ1 20
C26 + (-)-JQ1 50
C26 + (+)-JQ1 20
C26 + (+)-JQ1 50
e)
p-Beclin-1
p-Beclin-1/Beclin-1/vinculin (a.u.)
0.0
0.5
1.0
1.5
2.0
a**
a*
a*
b**
c*
b**
d*
Control
C26 + veh
C26 + (-)-JQ1 20
C26 + (-)-JQ1 50
C26 + (+)-JQ1 20
C26 + (+)-JQ1 50
75 -
75 -
100 -
p-Beclin-1
(Ser91/94)
Beclin-1
Vinculin
Control
C26+Veh
C26+(-)-JQ1 20
C26+(-)-JQ1 50
C26+(+)-JQ1 20
C26+J(+)-Q1 50

Supplementary Fig.5. JQ1 administration hampers the activation of proteolytic and autophagic transcripts in muscles from C26-tumor bearing mice

(**a**) Representative immunoblot showing slow Myosin Heavy Chain (sMyHC) protein expression in TA whole extracts from the 6 animal groups. Upper panel shows quantification of immunoblot bands from 4 animals per group. Data represent means±SD.

(**b-d**) Total RNA was extracted from TA muscles from control and C26 tumor-bearing mice treated with vehicle or JQ1 (-/+) and expression levels of Fbxo30/Musa1 (animals per group: n=10), LC3b (animals per group: n=5), ATG7 (animals per group: n=5) was measured by quantitative RT-PCR. Data represent means±SD.

(**e**) Representative Western blot of p-Beclin1 on TA whole extracts (animals per group: n=4). Bands quantifications are shown in the left panel. Data represent means±SD. *p<0.05; **p<0.01; ***p<0.001. "a" indicates statistical significance compared to Control; "b" indicates statistical significance compared to C26+vehicle; "c" indicates statistical significance compared to C26(-)-JQ1 20mg/kg/day; "d" indicates statistical significance compared to C26(-)-JQ1 50mg/kg/day. Statistical analysis was performed by using one-way ANOVA followed by Tukey's post-hoc test (a-e).

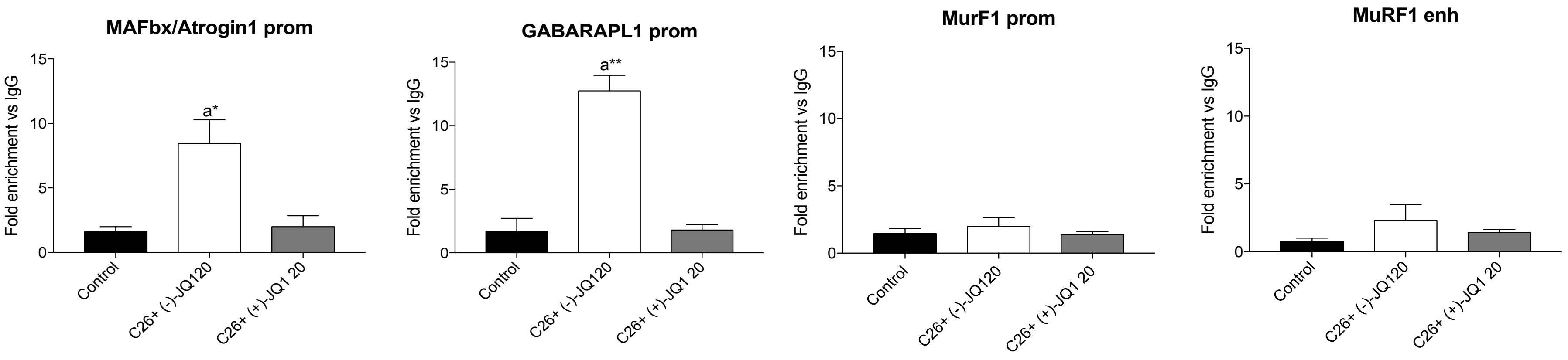

MAFbx/Atrogin1 prom
GABARAPL1 prom
MurF1 prom
MuRF1 enh
Fold enrichment vs IgG
0
5
10
15
a*
a**
Control
C26+ (-)-JQ120
C26+ (+)-JQ1 20

Supplementary Fig.6. BRD2 occupies MaFbx/Atrogin1 and GABARAPL1 promoters during cachexia and JQ1 administration prevents its recruitment

BRD2 ChIP qPCR at promoters of muscle catabolic genes. IgG was used as a reference. n=3. Data represent means±SEM. *$p<0.05$. Statistical analysis was performed by using one-way ANOVA followed by Tukey's post-hoc test. "a" indicates statistical significance compared to Control.

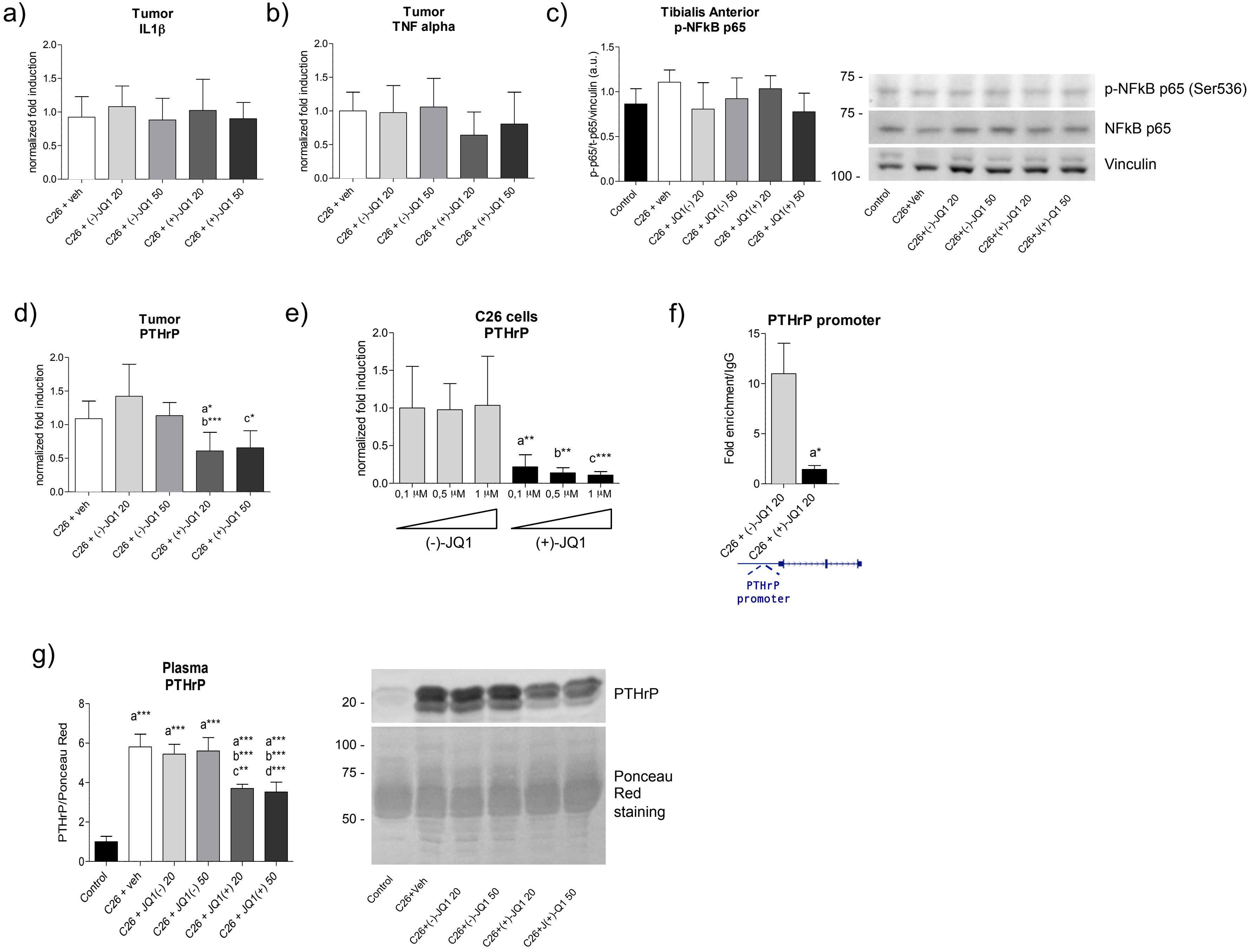
a)
Tumor
IL1β
normalized fold induction
C26 + veh
C26 + (-)-JQ1 20
C26 + (-)-JQ1 50
C26 + (+)-JQ1 20
C26 + (+)-JQ1 50
b)
Tumor
TNF alpha
normalized fold induction
c)
Tibialis Anterior
p-NFkB p65
p-p65/t-p65/vinculin (a.u.)
Control
C26 + veh
C26 + JQ1(-) 20
C26 + JQ1(-) 50
C26 + JQ1(+) 20
C26 + JQ1(+) 50
p-NFkB p65 (Ser536)
NFkB p65
Vinculin
Control
C26+Veh
C26+(-)-JQ1 20
C26+(-)-JQ1 50
C26+(+)-JQ1 20
C26+J(+)-Q1 50
d)
Tumor
PTHrP
normalized fold induction
a*
b***
c*
e)
C26 cells
PTHrP
normalized fold induction
a**
b**
c***
0,1 µM 0,5 µM 1 µM 0,1 µM 0,5 µM 1 µM
(-)-JQ1
(+)-JQ1
f)
PTHrP promoter
Fold enrichment/IgG
a*
C26 + (-)-JQ1 20
C26 + (+)-JQ1 20
PTHrP
promoter
g)
Plasma
PTHrP
PTHrP/Ponceau Red
a***
b***
c**
d***
Control
C26 + veh
C26 + JQ1(-) 20
C26 + JQ1(-) 50
C26 + JQ1(+) 20
C26 + JQ1(+) 50
PTHrP
Ponceau
Red
staining
Control
C26+Veh
C26+(-)-JQ1 20
C26+(-)-JQ1 50
C26+(+)-JQ1 20
C26+J(+)-Q1 50

Supplementary Fig.7. BRD4 regulates PTHrP expression in C26 tumors

(**a, b**) Total RNA was analyzed in tumors (n=8) from vehicle, (-)-JQ1 and (+)-JQ1 treated mice and IL1β, TNFα transcripts were measured by RT-PCR. (C) Representative Western blot of p-NFkB p65 (Ser 536) and total NFkB p65 levels, in TA whole extracts (animals per group: n=4).). Vinculin serves as loading control. Bands quantifications are shown in the left panel. Data represent means±SD.

(**d**) Total RNA was analyzed in tumors (n=8) from vehicle, (-)-JQ1 and (+)-JQ1 treated mice and PTHrP transcripts were measured by RT-PCR. *p<0.05, ***p<0.001. Data represent means ±SD. Statistical analysis was performed using one-way ANOVA followed by Tukey's post-hoc test. "a", "b", "c" indicates statistical significance compared to C26+vehicle, to C26+(-)-JQ1 20mg/kg/day and to C26+(-)-JQ1 50mg/kg/day respectively.

(**e**) C26 adenocarcinoma cells were treated with different doses of (-)-JQ1 and (+)-JQ1, total RNA was extracted and PTHrP transcript levels were measured by RTPCR. Data represent Mean±SD n=5. Statistical analysis was performed by using one-way ANOVA followed by Tukey's post-hoc test **p<0.01; ***p<0.001. "a", "b", "c" indicates statistical significance compared to C26 cells treated with (-)-JQ1 0.1µM, with (-)-JQ1 0.5µM and ed with (-)-JQ1 1µM respectively.

(**f**) BRD4 recruitment at PTHrP promoter was measured by ChIP experiments, in tumors from (-)-JQ1- and (+)-JQ1-treated (20mg/kg/day) animals (n=3 per experimental group). Data represent means±SEM. Statistical analysis was performed by Student's t-test *p<0.05.

(**g**) Representative Western blot showing PTHrP levels in plasma. Ponceau Red staining is shown as a loading control. Left panel: densitometric analysis. Animals per group: n=4. Data represent means±SD. Statistical analysis was performed by using one-way ANOVA followed by Tukey's post-hoc test. **p<0.01; ***p<0.001. "a", "b", "c", "d" indicates statistical significance compared to Control, to C26+vehicle, to C26(-)-JQ1 20mg/kg/day, to C26(-)-JQ1 50mg/kg/day respectively

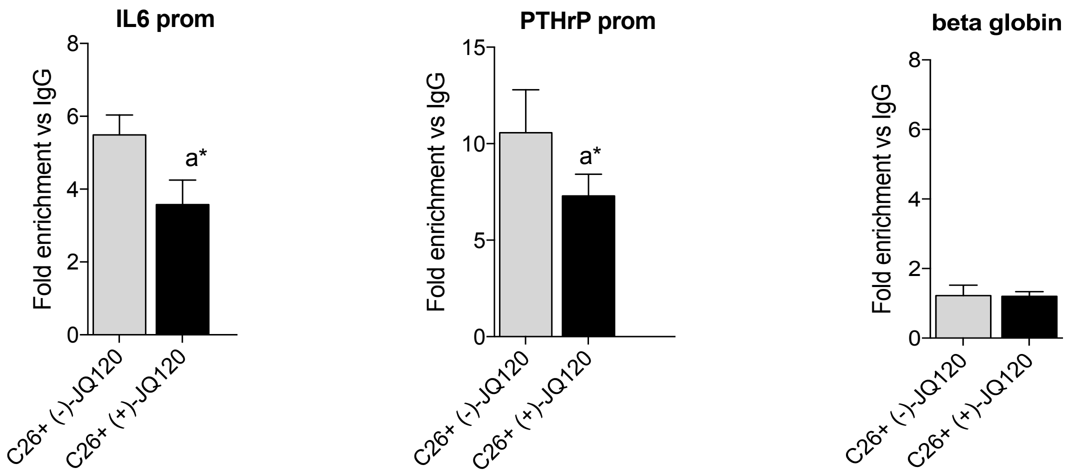

IL6 prom
Fold enrichment vs IgG
0
2
4
6
8
a*
C26+ (-)-JQ120
C26+ (+)-JQ120
PTHrP prom
Fold enrichment vs IgG
0
5
10
15
a*
C26+ (-)-JQ120
C26+ (+)-JQ120
beta globin
Fold enrichment vs IgG
0
2
4
6
8
C26+ (-)-JQ120
C26+ (+)-JQ120

Supplementary Fig.8. BRD2 associates with IL6 and PtHRP promoters in C26 tumors and JQ1 administration reduces its recruitment

BRD2 ChIP qPCR at IL6 and PtHRP promoters, in C26 tumors from vehicle- and (+)-JQ1-treated mice. IgG was used as a reference. n=3. Data represent means±SEM. Statistical analysis was performed by using Student's t-test *$p<0.05$. "a" indicates statistical significance compared to tumors obtained from vehicle treated mice.

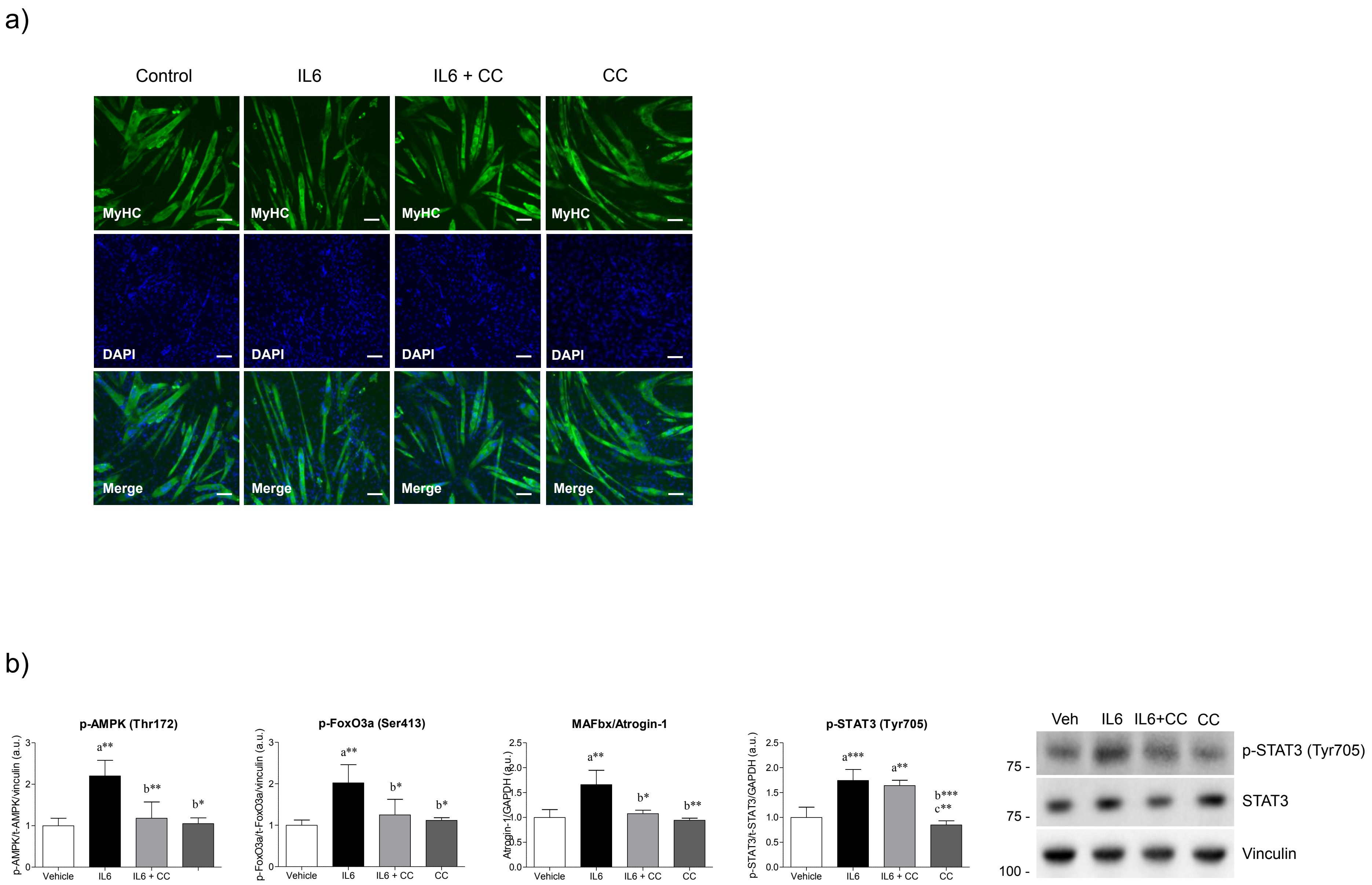
a)
Control
IL6
IL6 + CC
CC
MyHC
DAPI
Merge
b)
p-AMPK (Thr172)
p-AMPK/t-AMPK/vinculin (a.u.)
a**
b**
b*
Vehicle
IL6
IL6 + CC
p-FoxO3a (Ser413)
p-FoxO3a/t-FoxO3a/vinculin (a.u.)
a**
b*
b*
Vehicle
IL6
IL6 + CC
CC
MAFbx/Atrogin-1
Atrogin-1/GAPDH (a.u.)
a**
b*
b**
Vehicle
IL6
IL6 + CC
CC
p-STAT3 (Tyr705)
p-STAT3/t-STAT3/GAPDH (a.u.)
a***
a**
b***
c**
Vehicle
IL6
IL6 + CC
CC
Veh
IL6
IL6+CC
CC
p-STAT3 (Tyr705)
STAT3
Vinculin
75 -
75 -
100 -

Supplementary Fig.9. AMPK blockade with Compound C prevents IL6-induced atrophy in C2C12 myotubes

(**a**) Immunofluorescence in C2C12 myotubes with antibody against MyHC (green).DAPI was used to stain nuclei. C2C12 cells were allowed to differentiate in DM for 4 days and then treated with IL6 (20ng/ml) or IL6 (20ng/ml) and CompoundC (20μM) for 48 hours, and immunostained. Scale bar 50μm.

(**b**) Top panel: average normalized values of band intensity for immunoblots shown in Figure 5H. Bottom panel: densitometric analysis and representative Western blot of IL6- dependent STAT3 (Tyr705) phosphorylation in C2C12 myotubes treated as in Figure 5H. GAPDH was used as loading control. Data represent means±SD. Statistical analysis was performed by using one-way ANOVA followed by Tukey's post-hoc test. *$p<0.05$; **$p<0.01$. Statistical significance is expressed versus Vehicle.

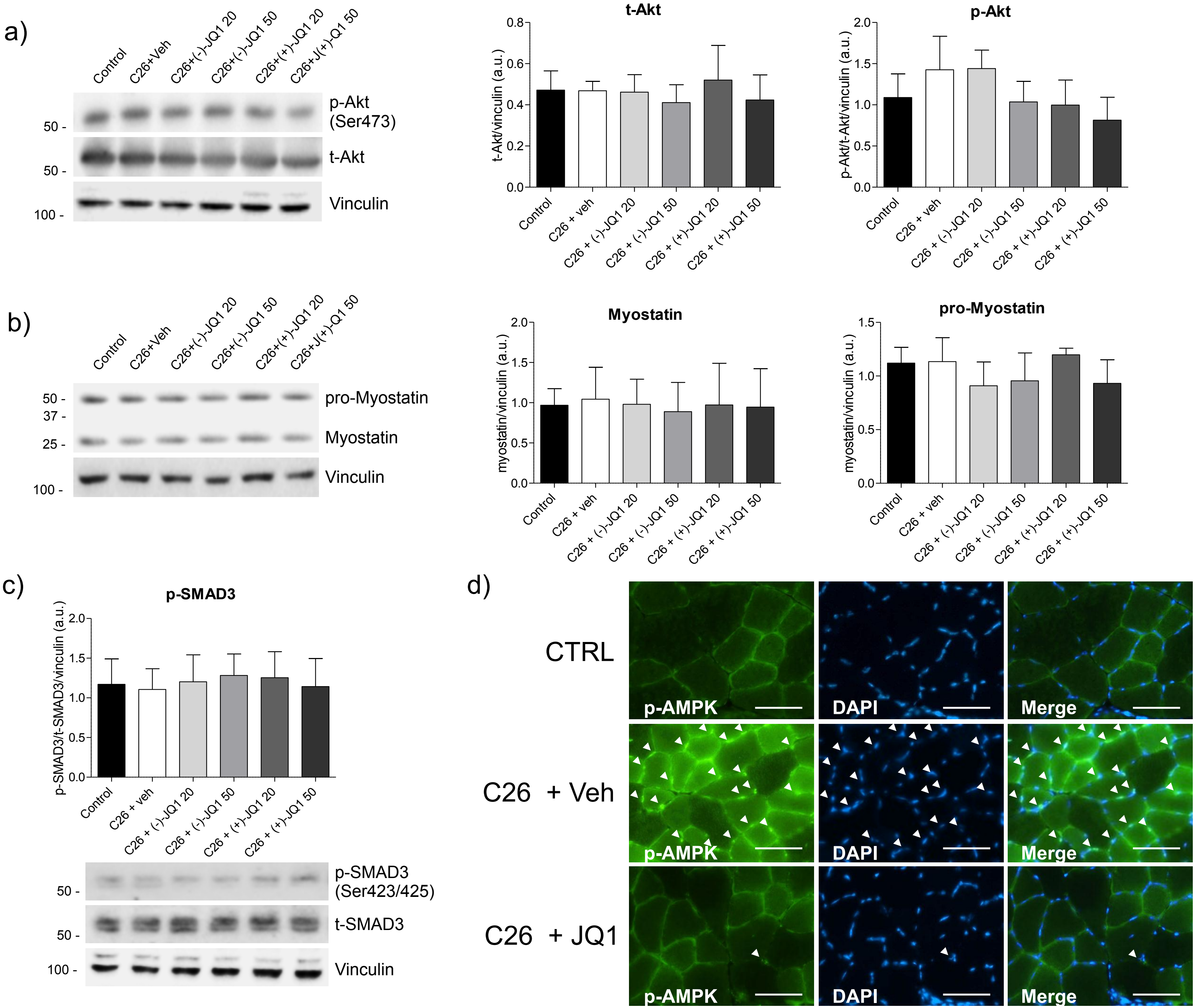

a)
Control
C26+Veh
C26+(-)-JQ1 20
C26+(-)-JQ1 50
C26+(+)-JQ1 20
C26+J(+)-Q1 50
50 -
50 -
100 -
p-Akt (Ser473)
t-Akt
Vinculin
t-Akt
t-Akt/vinculin (a.u.)
0.0
0.2
0.4
0.6
0.8
p-Akt
p-Akt/t-Akt/vinculin (a.u.)
0.0
0.5
1.0
1.5
2.0
Control
C26 + veh
C26 + (-)-JQ1 20
C26 + (-)-JQ1 50
C26 + (+)-JQ1 20
C26 + (+)-JQ1 50
b)
50 -
37 -
25 -
100 -
pro-Myostatin
Myostatin
Vinculin
Myostatin
myostatin/vinculin (a.u.)
pro-Myostatin
c)
p-SMAD3
p-SMAD3/t-SMAD3/vinculin (a.u.)
p-SMAD3 (Ser423/425)
t-SMAD3
d)
CTRL
C26 + Veh
C26 + JQ1
p-AMPK
DAPI
Merge

Supplementary Fig.10. Akt, Myostatin and SMAD3 phosphorylation is not altered by JQ1 treatment in in skeletal muscle of C26-tumor bearing mice

(**a**) p-Akt (Ser473) and total Akt levels were analyzed by Western blot in TA extracts of control and C26 tumor-bearing mice treated with vehicle, (-)-JQ1 and (+)-JQ1 (20 and 50 mg/kg/day). 4 animals were used for each experimental group. Left panel: representative Western blot. Right panels: p-Akt (Ser473) and total Akt bands were quantified and normalized with band intensity of vinculin. Data represent means±SD.

(**b**) Left panel: representative Western blot showing pro-Myostatin and the mature signaling peptide Myostatin in TA muscle lysates. Right panels: densitometric analysis of pro-Myostatin and Myostatin. Animals per group: n=4. Data represent means±SD.

(**c**) SMAD3 and p-SMAD3 (Ser423/425) levels were determined by Western blot analysis in TA muscles. 4 animals were used in each experimental condition. Upper panel: densitometric analysis of SMAD3(Ser423/425). Lower panel: representative Western blot. Data represent means±SD.

(**d**) Immunofluorescence was performed on frozen TA sections to analyze the expression and the distribution pattern of p-AMPK (Thr172) in control and C26 tumor-bearing mice treated with vehicle or (+)-JQ1 (20mg/kg/day). DAPI staining was used to visualize nuclei. Arrowheads indicate representative nuclei. Scale bar 50μm.

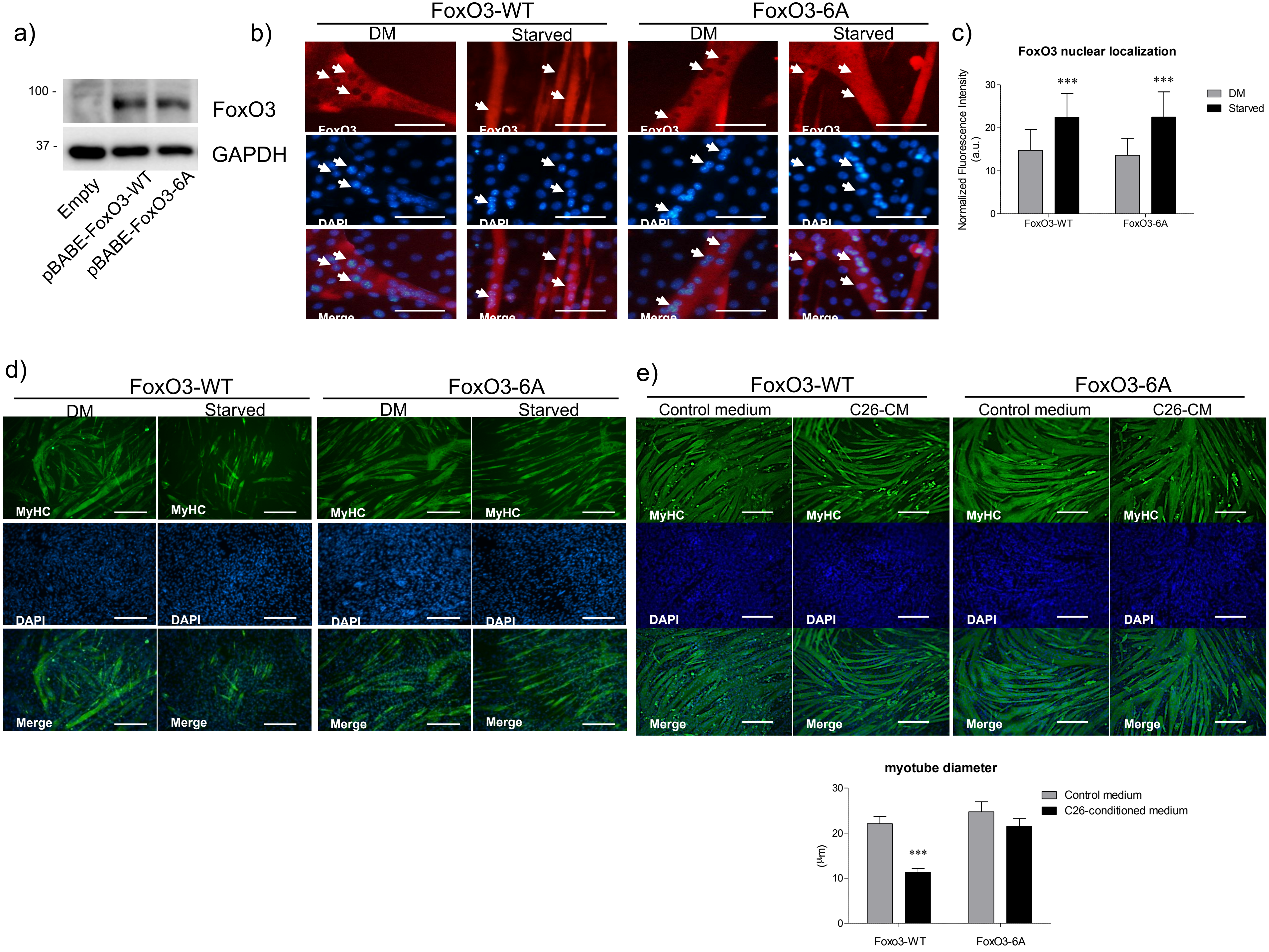
a)
100 -
37 -
FoxO3
GAPDH
Empty
pBABE-FoxO3-WT
pBABE-FoxO3-6A
b)
FoxO3-WT
FoxO3-6A
DM
Starved
FoxO3
DAPI
Merge
c)
FoxO3 nuclear localization
Normalized Fluorescence Intensity (a.u.)
DM
Starved
***
FoxO3-WT
FoxO3-6A
d)
FoxO3-WT
FoxO3-6A
DM
Starved
MyHC
DAPI
Merge
e)
FoxO3-WT
FoxO3-6A
Control medium
C26-CM
MyHC
DAPI
Merge
myotube diameter
(μm)
Control medium
C26-conditioned medium
***
Foxo3-WT
FoxO3-6A

Supplementary Fig.11. Mutations of AMPK phosphorylation sites do not affect FoxO3 nuclear localization during starvation

(**a**) C2C12 cells were transduced with pBabe-FoxO3-WT or pBabe-FoxO3-6A, and induced to differentiate in myotubes for 4 days. Western blot analysis was performed with an antibodies raised against FoxO3.

(**b**) pBabe-FoxO3-WT or pBabe-FoxO3-6A differentiated myotubes were starved in medium containing 0.5mM glucose and 0.1% horse serum for 24 hours, then fixed with paraformaldehyde and immunostained to visualize nuclear localization of FoxO3. DAPI was used for nuclei staining. Arrows show the absence/presence of FoxO3 nuclear localization in representative nuclei. Scale bar 25μm.

(**c**) Average of normalized fluorescence intensity of FoxO3 expressed as arbitrary units. 3 independent experiments were performed for each experimental condition. Data represent means±SD. . Statistical analysis was performed by using Student's unpaired t-test ***$p<0.001$

(**d**) C2C12 cells were transduced with pBabe-FoxO3-WT or pBabe-FoxO3-6A and allowed to differentiate in myotubes for 4 days. Cells were starved in 0.5mM glucose and 0.1% horse serum for 24 hours, fixed with paraformaldehyde and stained with antibody raised against MyHC (MF20). DAPI was used to visualize nuclei. Immunofluorescence images are representative of 3 independent experiments. Scale bar 50μm.

(**e**) C2C12 cells were transduced with pBabe-FoxO3-WT or pBabe-FoxO3-6A and allowed to differentiate in myotubes for 4 days. Cells were treated with C26-CM for 24 hours, fixed with paraformaldehyde and stained with antibody raised against MyHC (MF20). DAPI was used to visualize nuclei. Immunofluorescence images are representative of 3 independent experiments. Scale bar 50μm. Right panel: mean diameter of FoxO3-WT and FoxO3-6A myotubes is shown. Data represent means±SEM. Statistical analysis was performed by using one-way ANOVA followed by Tukey's post-hoc test . ***$p<0.001$ versus FoxO3-WT in DM.

Figure 5f

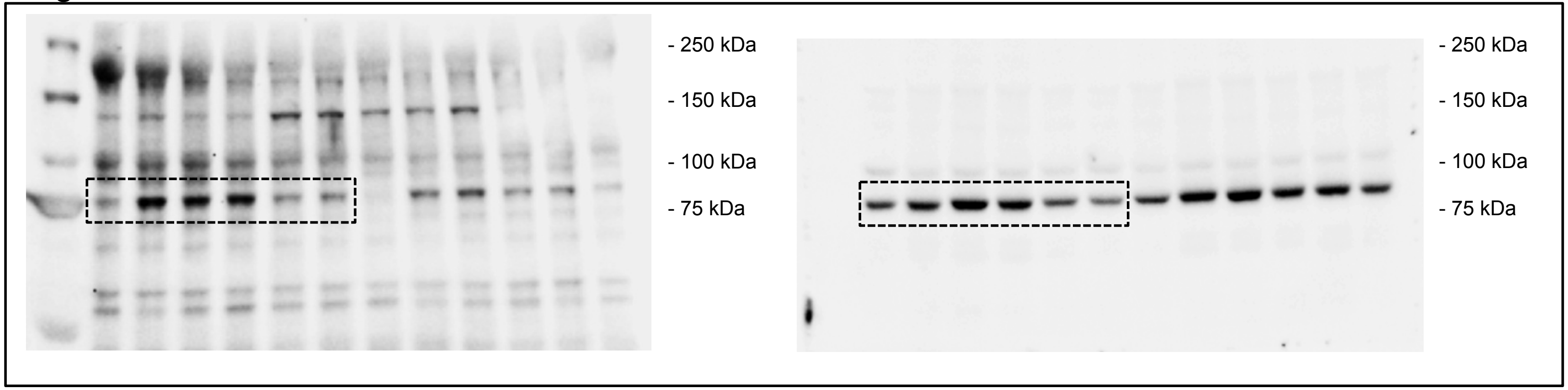

Figure 5f

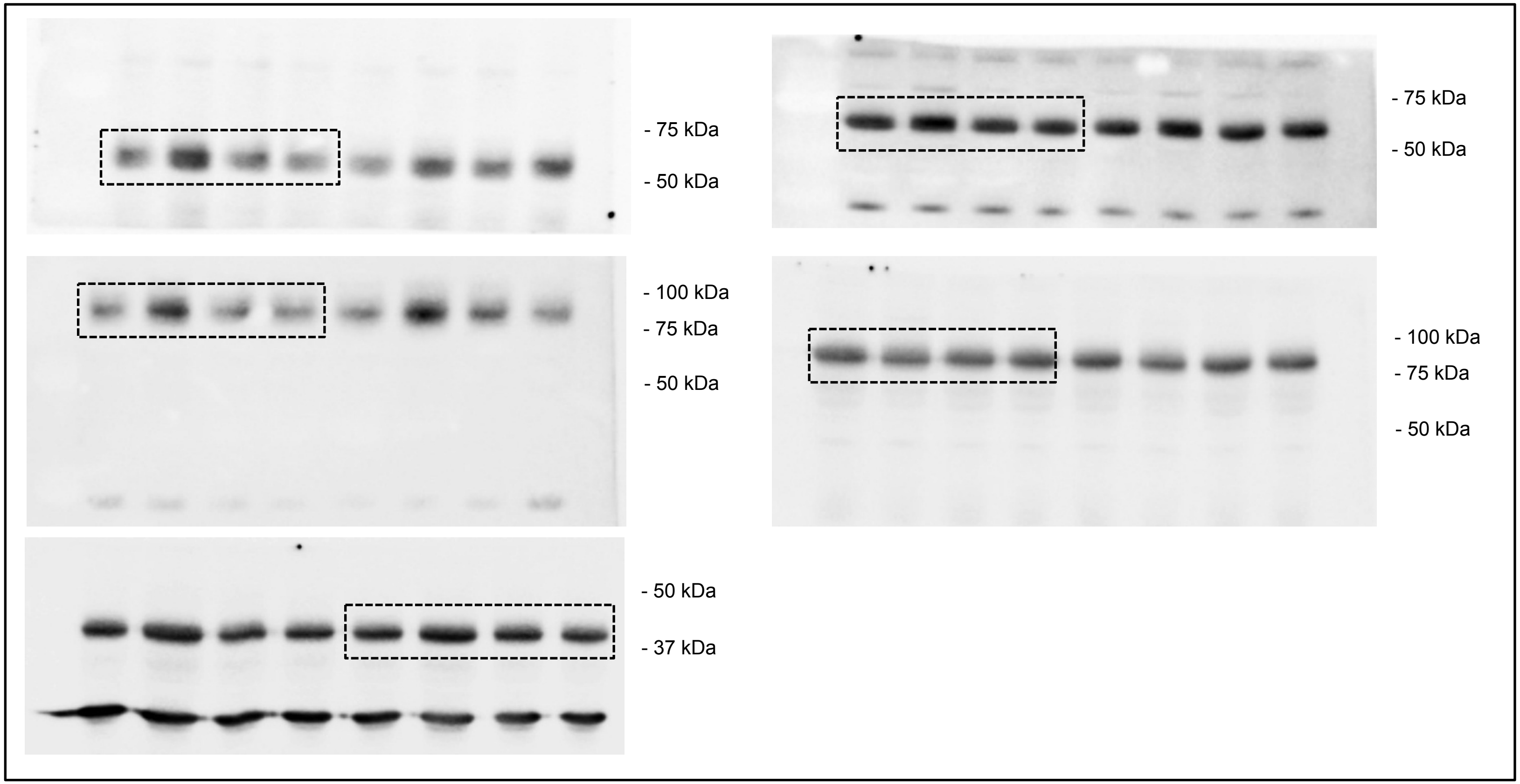

Figure 3b

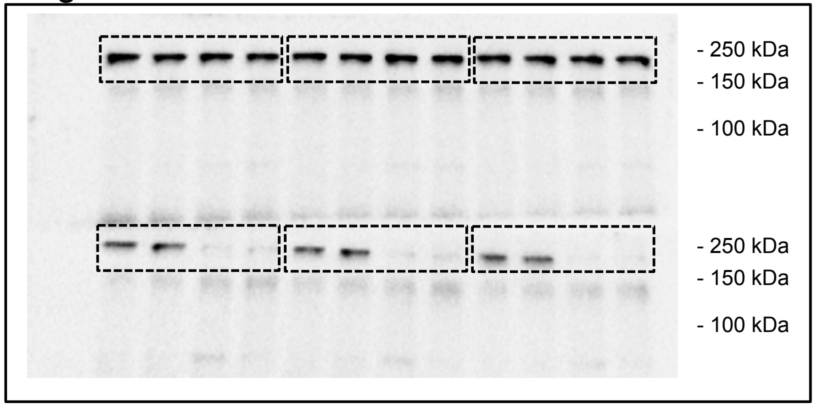

Figure 3e

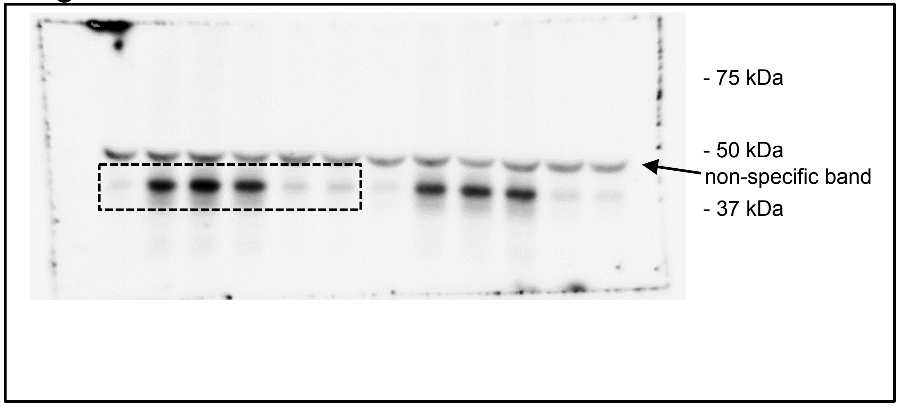

Figure 3i

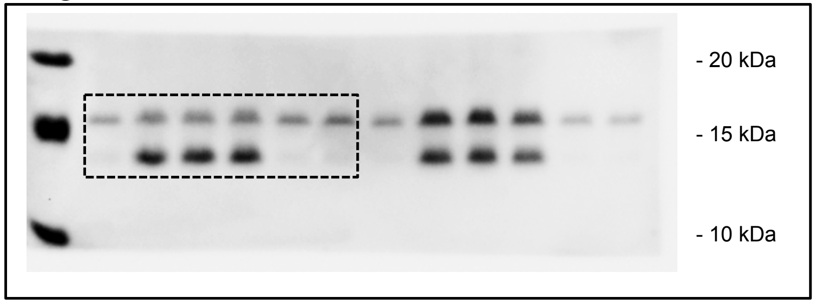

Figure 5c

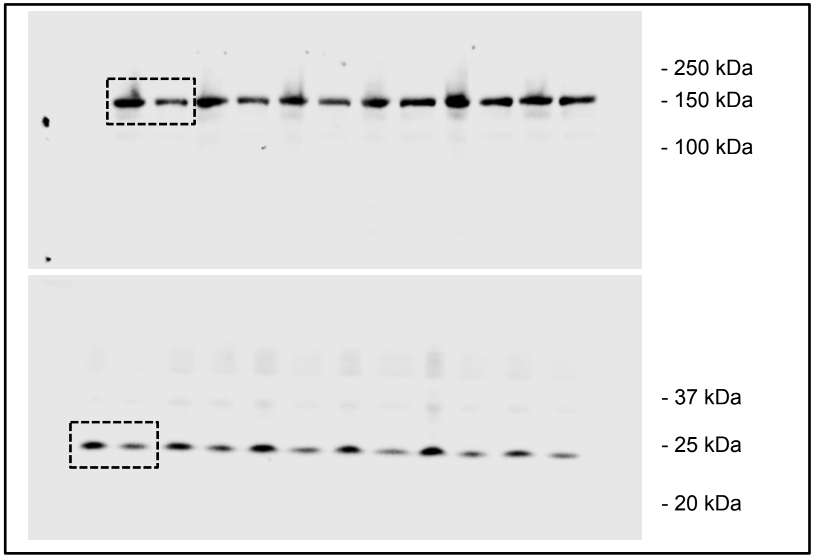

Figure 6a

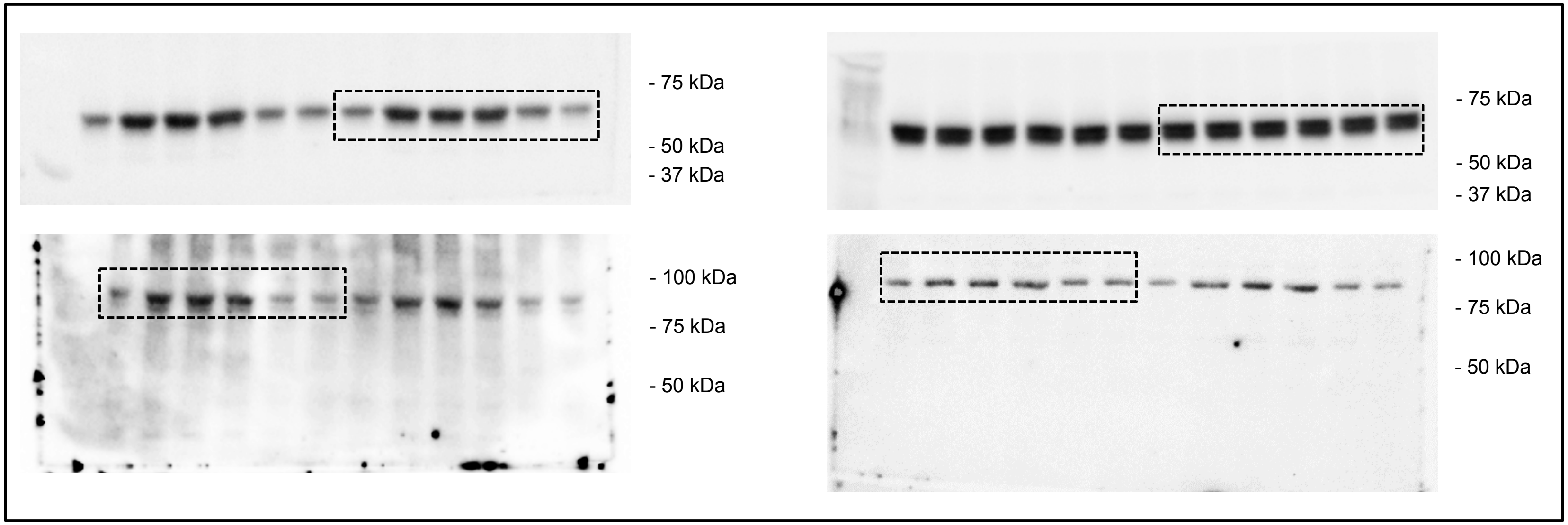

Figure 9b

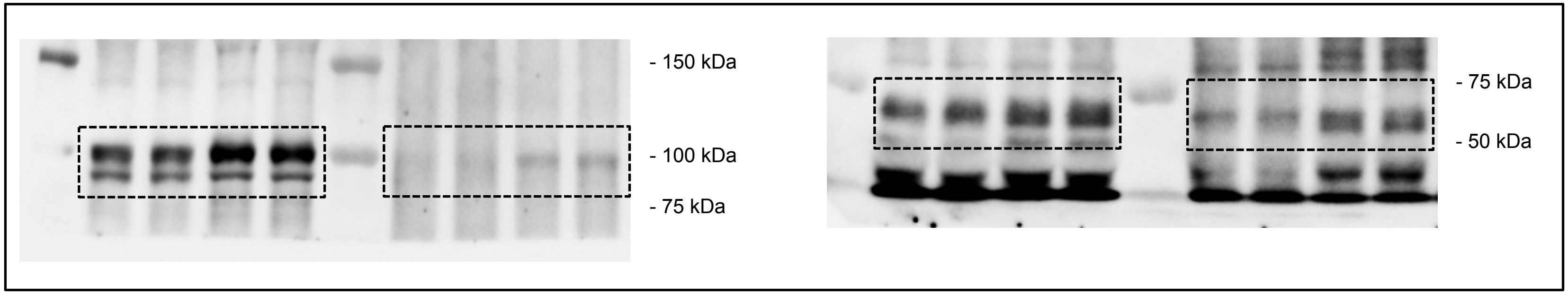

Supplementary Fig.12 Representative uncropped immunoblots.

Supplementary Table 1. Patients' data descriptor.

ID	CLASSIFICATION	AGE (years)	GENDER	BMI	WEIGHT LOSS (%)	TUMOUR SITE
C2	NN	74	F	26.3	0	-
C4	NN	81	F	23.2	0	-
C24	NN	62	M	24.7	0	-
C28	NN	63	M	23.9	1.51	-
C31	NN	61	M	23.1	0	-
C34	NN	71	M	23.7	0	-
C36	NN	68	M	24.6	0	-
C37	NN	61	M	22.7	0	-
K3	CC	76	M	28.3	7.22	COLON
K24	CC	88	M	21.5	6.06	COLON
K50	CC	51	F	22.6	11.76	COLON
K68	CC	77	F	19.5	5.66	COLON
K73	CC	74	F	22.89	5.17	COLON
K11	CC	58	M	25.1	12.5	PANCREAS
K13	CC	67	M	19.7	6.56	PANCREAS
K33	CC	58	M	21.5	13.89	PANCREAS
K57	CC	61	M	20	25	PANCREAS
K69	CC	67	M	19.7	13.64	PANCREAS
K34	CC	63	F	20	24.24	PANCREAS
K38	CC	55	F	30.6	5.56	PANCREAS
K39	CC	66	F	24.7	10.29	PANCREAS
K40	CC	56	F	18.9	8.77	PANCREAS
K48	CC	59	F	25.8	15.4	PANCREAS

NN: Non Neoplastic patient
CC: Cachectic Cancer patient

Supplementary Table 2. List of Primers used in quantitative RT-PCR analysis

GENE	SEQUENCE
ACCα	GCCTCTTCCTGACAAACGAG<br>GGTCCCTGCTTGTCTCCATA
ATG7	GTTTCTGCTCCTGACCTTCG<br>GGGATGCTCTCAGGAAG ACA
Atrogin/MAFbx	CACATTCTCTCCTGGAAGGGC<br>TTGATAAAGTCTTGAGGGGAA
Bnip3	GCGAGAAAAACAGCACTCTG<br>TTCCCCCTTTCTTCATAACG
Brd4	CCTCCCAGTGTGCCCCTTCTT<br>CTGAGTCGGAGAGCACCAGCG
Cathepsin L	GTGGACTGTTCTCACGCT CAAG<br>TCCGTCCTTCGCTTCATA GG
Fas	GCTGGCATTCGTGATGGAGTCGT<br>AGGCCACCAGTGATGATGTAACTCT
Fbxo30/MUSA1	GCAGTGGGGGAAGAAGAAGT<br>AGCCATGCTCAGGATGT CAG
FoxO3a	CTCGTGGAAGGGAGGAGGAGGAAT<br>CCTTCAGGAACGAGGCGGGA
Gabarapl1	CATCGTGGAGAAGGCTCCTA<br>TCCTCAGGTCTCAGGTGGAT
GAPDH	AACATCAAATGGGGTGAGGCC<br>GTTGTCATGGATGACCTTGGC

IL 1beta	GCCACCTTTTGACAGTGATGAG<br>GCCACCTTTTGACAGTGATGAG
IL-6	GCCAGAGTCCTTCAGAGAGA<br>TGGTCCTTAGCCACTCCTTC
LC3b	GTCCGAGAAGACCTTCAAGC<br>AAGCGCCGTCTGATTATCTT
Murf-1	AGTGTCCATGTCTGGAGGT<br>AATGATGTTTTCCACCAGC
Socs3	GCAAGCTGCAGGAGAGCGGATT<br>AAGAAGTGGCGCTGGTCCGA
TNF alpha	CTGTAGCCCACGTCGTAGC<br>TTGAGATCCATGCCGTTG
PThrP	TGGTTCAGCAGTGGAGTGTC<br>GGATGGACTTGCCCTTGTC

Supplementary Table 3. List of primers used in ChIP analysis

GENE	SEQUENCE
Atrogin promoter	GGGACAAGAGTGGGTCA ACTA<br>CAGCATTCCCAGAGTCA GGAG
GABARAPL1 promoter	ATAAACAAAGCTTCTGTCCACCC<br>AGAGCTGGAAACACAAAAACACC
IL-6 promoter	AATAGTCCTTCCTACCCCAATTTC<br>ATTTCAAGATGAATTGGATGGTCT
Murf-1 promoter	CCTGCATGTGATCTGAGAGG<br>CCCGACTTCTGTCTTGGTCT
PThrP promoter	CTCTTTGCGACTCGCTCACT<br>GCAGGTTGGAGAGTAGCTGTG
beta – globin promoter	GACAAACATTATTCAGAGGGAGT<br>AAGCAAATGTGAGGAGCAACTGAT

Supplementary Table 4. List of antibodies used in this study.

Antibody	Ref	Provider	Application and Dilution
Actin	A2066	SIGMA	WB (1:3000)
ACCα	sc-30212	Santa Cruz	WB (1:1000)
p-ACCα (Ser 79)	sc-271965	Santa Cruz	WB (1:1000)
AMPKα1/2	sc-25792	Santa Cruz	WB (1:3000)
p-AMPKα1/2 (Thr 172)	sc-33524	Santa Cruz	WB (1:1000), IF (1:50),ChIP (3μg)
Akt1/2/3 (H136)	sc-8312	Santa Cruz	WB (1:1000)
p-Akt1/2/3 (Ser 473)	sc-7985-R	Santa Cruz	WB (1:1000)
ATGL	NBP1-25852	Novus Bio	WB (1:2000)
Atrogin MAFbx (H-300)	Ab-168372	Abcam	WB (1:3000)
Beclin	Sc-10086	Santa Cruz	WB (1:1000)
p-Beclin (Ser91/94)	#12476	Cell Signalling	WB (1:400)
BRD4	#13440	Cell Signalling	ChIP-Seq (5μg), ChIP (3μg)
BRD4	sc-48772	Santa Cruz	WB (1:1000)
BRD2	A302-583A	Bethyl	ChIP (3μg)
BRD2	cs-5848s	Cell signaling	ChIP (3μg)
c-Myc A14	sc-789	Santa Cruz	WB (1:500)
Caspase-3	sc-7148	Santa Cruz	WB (1:1000)
FoxO3a	sc-11351	Santa Cruz	WB (1:1000),ChIP (5μg),IF (1:100)
p-FoxO3a (Ser253)	#9466s	Cell Signalling	WB (1:1000)
p-FoxO3a (Ser413)	#8174S	Cell Signalling	WB (1:1000), IF (1:50)
GAPDH	sc-32233	Santa Cruz	WB (1:3000)
H3	sc-10809	Santa Cruz	WB (1:1000)

H3 Ser10-p	ab5176	Abcam	WB (1:2000)
IL-6 (D5W4V)	#12912S	Cell Signalling	WB (1:1000)
IgG	sc-2027	Santa cruz	ChIP (3μg)
LC3b	L7543	SIGMA	WB (1:2000)
MyHC MF20	DSHB	MF 20	IF (1:30)
slow MyHC	m8421	SIGMA	WB (1:5000)
fast MyHC	m4276	SIGMA	WB (1:5000)
Myostatin GDF-8	sc-6885-R	Santa Cruz	WB (1:1000)
NF-KB P65	SC-372	Santa Cruz	WB (1:2000)
P-NFKB p65	sc-33020	Santa Cruz	WB (1:1000)
PTH-rP (H-137)	sc-20728	Santa Cruz	WB (1:1000)
SREBP-1 (H-160)	sc-8984	Santa Cruz	WB (1:1000)
Smad3 (C67H9)	#9523S	Cell Signalling	WB (1:500)
p-Smad3 (C25A9)	#9520S	Cell Signalling	WB (1:500)
Stat3 (C-20)	sc-482	Santa Cruz	WB (1:2000)
p-Stat3 (Tyr705) (D3A7)	#9145s	Cell Signalling	WB (1:1000)
RNA PolII, clone 8WG16	Ab817	Abcam	ChIP (3μg)
ULK1(H240)	sc-33182	Santa Cruz	WB (1:500)
p-ULK 1 (Ser 556)	TA310925	Origene	WB (1:300)
Vinculin	V9264	SIGMA	WB (1:10,000)
Goat anti-Mouse IgG 488	A11029	Thermo Scient.	IF (1:500)
Goat anti-Rabbit IgG 568	A11011	Thermo Scient.	IF (1:500)
Goat anti-Rabbit IgG 488	A11008	Thermo Scient.	IF (1:500)
